# Haem-iron plays a key role in the regulation of the Ess/type VII secretion system of *Staphylococcus aureus* RN6390

**DOI:** 10.1099/mic.0.000579

**Published:** 2017-11-24

**Authors:** M. Guillermina Casabona, Holger Kneuper, Daniela Alferes de Lima, Catriona P. Harkins, Martin Zoltner, Erik Hjerde, Matthew T. G. Holden, Tracy Palmer

**Affiliations:** ^1^​ Division of Molecular Microbiology School of Life Sciences, University of Dundee, Dundee, UK; ^2^​ School of Medicine, University of St Andrews, St Andrews KY16 9TF, UK; ^3^​ Department of Chemistry, Arctic University of Norway, Tromsø, Norway

**Keywords:** *Staphylococcus aureus*, protein secretion, iron homeostasis, RNA-sequencing

## Abstract

The *Staphylococcus aureus* type VII protein secretion system (T7SS) plays important roles in virulence and intra-species competition. Here we show that the T7SS in strain RN6390 is activated by supplementing the growth medium with haemoglobin, and its cofactor haemin (haem B). Transcript analysis and secretion assays suggest that activation by haemin occurs at a transcriptional and a post-translational level. Loss of T7 secretion activity by deletion of *essC* results in upregulation of genes required for iron acquisition. Taken together these findings suggest that the T7SS plays a role in iron homeostasis in at least some *S. aureus* strains.

## Introduction

Bacteria produce a number of different secretion machineries to transport proteins across their cell envelopes [1]. Secreted proteins play essential roles in environmental adaptation and in pathogenic bacteria are frequently linked with the ability to cause disease. The type VII protein secretion system (T7SS) was discovered almost 15 years ago in pathogenic *Mycobacteria*. This system, also termed ESX-1, was shown to secrete two small proteinaceous T-cell antigens and to be essential for virulence [2–4].

*Mycobacteria* produce up to five different T7SSs [5, 6]. In addition to ESX-1, ESX-5 also plays a key role in host interaction during pathogenesis [7]. Of the other ESX systems in *Mycobacteria*, ESX-3 is the best studied and is critical for siderophore-mediated acquisition of iron [8–10]. Consistent with their diverse roles in the physiology and virulence of *Mycobacteria*, the ESX systems are differentially regulated. For example, expression of ESX-1 is under indirect transcriptional control of the PhoPR two-component system [11] that appears to respond to low pH, conditions that are found in phagolysosomes [12]. ESX-5 expression is induced in response to phosphate starvation [13] whilst ESX-3 expression is de-repressed when cells are starved of iron or zinc [14, 15].

T7SSs are also found in other bacteria, in particular from the Gram-positive low G+C phylum *Firmicutes* [16]. Similarity between the T7SS found in *Firmicutes* and the well-studied mycobacterial ESX T7SSs is limited, with the systems sharing only two common types of components. This has resulted in the T7SS of *Firmicutes* such as *Staphylococcus aureus* being designated Ess or T7b to distinguish them from the better-characterized mycobacterial T7a systems [17]. One of the components shared between the two systems is a membrane-bound ATPase of the FtsK/SpoIIIE family termed EccC (T7a) or EssC (T7b). This component forms a hexameric assembly that probably acts as the motor protein and potentially also the translocation channel of the T7SS [18, 19]. The second common component is at least one small protein of the WXG100 family, EsxA, which is secreted by the T7SS. In *Mycobacteria*, EsxA homologues are secreted as heterodimers with EsxB partner proteins (e.g. [20, 21]) whereas in *Firmicutes* EsxA is secreted as a homodimer [22].

The T7SS of *S. aureus* is encoded by the *ess* locus. In addition to EsxA and EssC, four further proteins encoded by the locus – EsaA, EssA, EssB and EsaB – are essential components of the secretion machinery [23–25]. The *ess* locus is under complex transcriptional control by the alternative sigma factor σ^B^ and expression is also repressed by the two-component SaeSR system [26, 27]. Experiments using mouse models of infection have indicated that the Ess system is required for virulence, in particular for the persistence of abscesses in the liver and kidney [23–25, 28]. It is also required for colonization and for intraspecies competition [25, 29]. The secretion system appears to be highly expressed in mammalian hosts [30], and in at least one strain is transcriptionally activated by pulmonary surfactant [31]. However, in laboratory growth media, although the secretion system components are produced, the machinery is poorly active and the levels of secreted substrates are relatively low [25, 29, 32].

In this study we have attempted to identify factors that activate secretion by the T7SS *in vitro*. We show that addition of haemin (haem B) enhances T7 secretion in at least two different *S. aureus* strains. Moreover, we also show that in the absence of a functional T7SS, the laboratory strain RN6390 upregulates numerous genes involved in iron acquisition. Together our findings point to a potential role of the T7SS in *S. aureus* iron homeostasis.

## Methods

### Bacterial strains and growth conditions for secretion assays

The *S. aureus* strains used in this study are listed in Table 1. *S. aureus* strains were grown overnight at 37 °C with shaking in tryptic soy broth (TSB). To test *S. aureus* growth with various media additives, strains were subcultured in either TSB or RPMI (Sigma) as indicated, supplemented with the corresponding additives. Additives were made fresh and sterilized by filtration, and were dissolved in distilled water except for the haemin and other protoporhyrins, which were dissolved in DMSO, and haemoglobins, which were dissolved in 0.1 M NaOH. For secretion assays, the indicated strains were grown to an OD_600_ of 2 and fractionated to give whole cell lysates and supernatant fractions as described previously [25]. Chelation of divalent cations from TSB was achieved after a 30 min treatment with 5 % Chelex-100 (BioRad). Chelex-treated TSB was then supplemented with 25 µM ZnCl_2_, 25 µM MnCl_2_, 1 mM MgCl_2_ or 100 µM CaCl_2_ [33].

**Table 1.** *S. aureus* strains used in this study MRSA, methicillin-resistant *S. aureus*

**Strain**	**Relevant genotype or description**	**Source or reference**
RN6390	NCTC8325 derivative, *rbsU*, *tcaR*, cured of φ11, φ12, φ13	[54]
COL	Healthcare acquired MRSA (HA-MRSA)	[55, 56]
USA300	Community-acquired MRSA (CA-MRSA)	[57]
10.1252.X	ST398-like isolate. Livestock-associated	Roslin Institute, Edinburgh, UK
MRSA252	HA-MRSA, representative of Epidemic MRSA-16	[58]
HO 5096 0412	HA-MRSA, representative of Epidemic MRSA-15	[59]

### Preparation of a polyclonal EsxB antibody

The EsxB (UniProt accession Q99WT7) coding sequence was PCR-amplified from a synthetic gene (codon optimized for *Escherichia coli* K12; Genscript) using the forward primer 5′-GCGCGTCGACAATGGGCGGCTATAAAGG C-3′ and the reverse primer 5′-GCGCCTCGAGTTACGGGTTCACGCGATCCAGGC-3′, and cloned into the *Sal*I/*Xho*I site of a modified pET27b vector (Novagen). The plasmid produces an N-terminal His_6_-tagged protein with a TEV (tobacco etch virus) protease cleavage site. The protein was expressed and purified as described previously [34], except that in the final size exclusion chromatography step an HR 30/100 GL Superdex75 column (column volume=24 ml; GE Healthcare) equilibrated with 20 mM Tris pH 7.8 and 100 mM NaCl was used. Two milligrams of purified EsxB (retaining a Gly–Ala–Ser–Thr sequence at the N terminus after the cleavage step) was utilized as antigen to immunize rabbits for polyclonal antibody production in a standard three-injection protocol (Seqlab).

### Western blotting and protein quantification

Samples were mixed with LDS loading buffer (NuPAGE LDS sample buffer) and boiled for 10 min prior to separation on bis-Tris gels. Western blotting was performed according to standard protocols with the following antibody dilutions: α-EsxA [25] 1 : 2500, α-EsxB 1 : 1000, α-EsxC [25] 1 : 2000, α-TrxA [35] 1 : 25 000. Horseradish peroxidase-conjugated secondary antibodies (Bio-Rad) were used as per the manufacturer’s instructions. For protein quantification, western blots of at least three biological replicates were developed with chemiluminescence (Clarity Western ECL Blotting Substrate; BioRad) and light-emitting bands were visualized with a CCCD camera (GeneGNOME XRQ; Syngene). Densitometry analysis was performed using ImageJ [36].

### RNA isolation and qPCR

For the RNA-seq analysis, three biological repeats of the indicated *S. aureus* strains were grown aerobically in TSB up to an OD_600_ of 1 at which point mRNA was prepared (in three technical replicates). Total mRNA was extracted, reverse transcribed and sequenced as described previously [37]. The sequence reads from each individual dataset were mapped to the *S. aureus* NCTC 8325 genome using EDGE-pro [38], and quantification of transcript abundance and calculation of differential gene expression were performed using DEseq2 [39]. DEseq2 uses the Negative Binomial distribution as a model to compute *P*-values, and we regarded *P*>0.05 as the probability of observing a transcript's expression levels in different conditions by chance. Reads were aligned using the Tophat aligner [40] and to acquire a single transcriptome for each strain, the three assemblies produced by cufflinks were merged and the abundances of each sample were assembled using cuffquant. Differential expression was analysed using edgeR [41]. Genes were considered to be differentially expressed when log fold change was >2 or <−2 and the q value <0.05. The RNA-seq data from this study were submitted to the European Nucleotide Archive with accession number ERP009279 and in Array express under accession number E-ERAD-362.

To isolate mRNA for RT-PCR, three biological repeats of the indicated *S. aureus* strains were grown aerobically in TSB in the presence or absence of 1 µM haemin up to an OD_600_ of 1, at which point mRNA was prepared. Total mRNA was extracted using the SV Total RNA Isolation Kit (Promega) with modifications as described by Kneuper *et al*. [25]. Briefly, cell samples were stabilized in 5 % phenol/95 % ethanol on ice for at least 30 min and then centrifuged at 2770 ***g*** for 10 min. Cells were then resuspended in 100 µl of TE buffer containing 500 µg lysostaphin ml^−1^ and 50 µg lysozyme ml^−1^ and incubated at 37 °C for 30 min. Subsequently, the manufacturer’s instructions were followed and the isolated RNA was subjected to a second DNase treatment using a DNA-free kit (Ambion). RNA was stored at −80 °C until use. To determine transcript levels, 500 ng of cDNA template was used with the following primer pairs: *esxA* (5′-TGGCAATGATTAAGATGAGTCC-3′ and 5′-TCTTGTTCTTGAACGGCATC-3′ [25]), *esxC* (5′-AAGCATGCTGAAGAGATTGC-3′ and 5′-TCTTCACCCAACATTTCAAGC-3′) and 16S rRNA (5′-GTGCACATCTT GACGGTACCTA-3′ and 5′-CCACTGGTGTTCCTCCATATC-3′ [25]). Quantitative PCR was performed using a thermal cycler. Three technical replicates were prepared for each culture condition, using 2* Quantifast SYBR Green PCR master mix (Qiagen) according to the manufacturer’s instructions. Standard curves were generated from serial 10-fold dilutions of genomic DNA. Amplification results were analysed with MxPro QPCR software (Stratagene) to give the levels of mRNA normalized to the level of 16S rRNA amplification in each sample. Results were further analysed in Microsoft Excel to calculate relative expression levels.

### Construction of an *esxA-yfp* transcriptional/translational fusion

The *yfp* gene was amplified without its start codon using Yfpfuse1 (5′-GGAACTACTAGATCTTCAAAAGGC-3′) and Xfpfuse2 (5′-CAAATAAGAATTCTGAGCGCCGG-3′) and cloned as a *Bgl*II/*Eco*RI fragment into pRMC2 [42], generating pRMC2-*yfp*. An approximately 500 bp region covering the *esxA* promoter, ribosome binding site and start codon was amplified using primers esxprom1 (5′-GAATGGTACCGATTGTTGTTAAGATC-3′) and esxprom2 (5′-TTAGATCTTGCCATAACTAGAAACC-3′) with RN6390 chromosomal DNA as template, and cloned as a *Kpn*I/*Bgl*II fragment into pRMC2-*yfp* to give plasmid pPesxA-yfp.

## Results and Discussion

### T7SS in strain RN6390 is stimulated by supplementation with calcium ions, haemoglobin and haemin

**Fig. 1.**
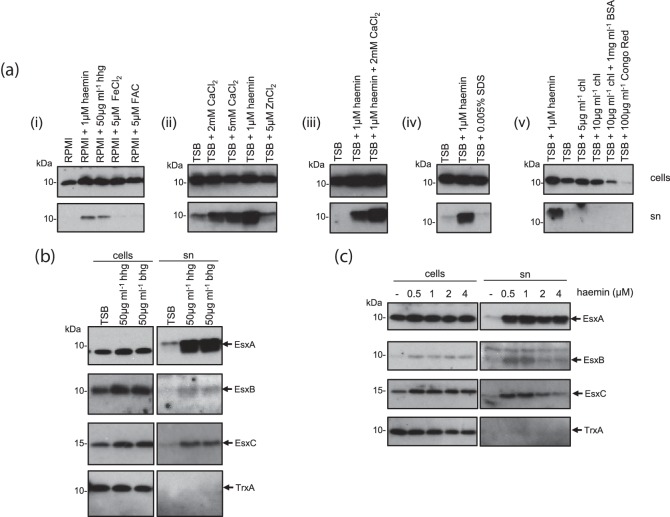
T7 secretion in strain RN6390 is stimulated by haemoglobin, haemin and millimolar concentrations of CaCl_2_. RN6390 was subcultured into either RPMI or TSB media, supplemented with the indicated additives, and grown aerobically until an OD_600_ of 2 was reached. Samples were fractionated to give cells and supernatant (sn), and supernatant proteins were precipitated using TCA. For each gel, 4 µl of a cell culture adjusted to an OD_600_ of 1 and 12 µl of culture supernatant were loaded. Final concentrations of additives are as indicated. hhg, Human haemoglobin; bhg, bovine haemoglobin; FAC, ferric ammonium citrate; chl, cholesterol; BSA, bovine serum albumin. (a) Western blots were probed with anti-EsxA antisera. (b and c) Western blots were probed with anti-EsxA, anti-EsxB, anti-EsxC or anti-TrxA (cytoplasmic control) antisera.

Protein secretion systems are frequently activated in a post-translational manner; for example, type III secretion is activated by addition of the amphipathic dye Congo Red, or by calcium deprivation [43, 44] and the type VI secretion system is activated by protein phosphorylation [45]. We therefore sought to determine whether we could activate secretion by the T7SS in our standard laboratory strain of *S. aureus*, RN6390, by making empirical additions to the growth media. As shown in Fig. 1(a), panel (i), some secretion of the T7 core component, EsxA, could be detected when the strain was grown in either RPMI or TSB growth media. In general we noted that more EsxA was detected in the supernatant after growth in TSB than in RPMI (Fig. 1a).

### Haemoglobin and haemin stimulate T7 secretion

Both TSB and RPMI lack an exogenously added iron source, and RPMI is considered to be iron-limited [46]. We therefore first tested the effect of exogenous iron sources on EsxA secretion. It can be seen that haemoglobin had a strikingly positive effect on EsxA levels in the culture supernatant for RN6390 grown both in RPMI and in TSB media (Fig. 1a, panel i; Fig. 1b). Fig. 1(b) confirms that secreted levels of the T7 substrate proteins EsxB and EsxC [23, 24] were also similarly enhanced in the presence of haemoglobin. We tested whether other iron sources could also stimulate T7 secretion. Fig. 1(a) (panel i) shows that neither ferric ammonium citrate (FAC) nor ferrous chloride stimulated EsxA secretion in RPMI, indicating that it was not a general effect of increased iron availability. The mycobacterial ESX-3 T7SS is transcriptionally regulated by both iron and zinc [14, 15]. However, supplementation of the growth medium with 5 µM zinc had no detectable effect on EsxA secretion (Fig. 1a, panel ii).

We next tested whether the iron-containing cofactor component of haemoglobin, haemin (haem B), could also enhance EsxA secretion. Fig. 1(a) shows that supplementation of both RPMI (panel i) and TSB media (Fig. 1a, panels ii–v) with 1 µM haemin resulted in a marked increase in EsxA secretion. We confirmed that haemin had a similar stimulatory effect on secretion of the T7 substrates EsxB and EsxC (Fig. 1c). Fig. 1(c) also shows that EsxA, EsxB and EsxC secretion were enhanced to similar levels in the presence of 0.5 and 1 µM haemin but at higher haemin concentrations secretion was reduced. We conclude that haemoglobin and its cofactor, haemin, can positively regulate T7 secretion.

**Fig. 2.**
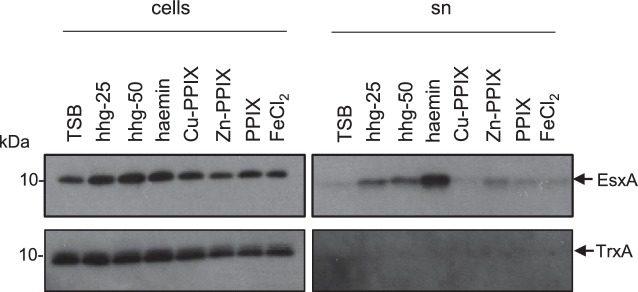
Stimulation of EsxA secretion is specific to the Fe-loaded PPIX. RN6390 was subcultured into TSB medium supplemented with the indicated additives, and grown aerobically until an OD_600_ of 2 was reached. Samples were fractionated to give cells and supernatant (sn), and supernatant proteins were precipitated using TCA. For each gel, 4 µl of a cell culture adjusted to an OD_600_ of 1 and 12 µl of culture supernatant were loaded. Human haemoglobin (hhg) was added at a final concentration of either 25 or 50 µg FeCl_2_ ml^−1^ at 5 µM and all other supplements at 2 µM. PPIX, protoporphyrin IX. Western blots were probed with anti-EsxA or anti-TrxA (cytoplasmic control) antisera.

To determine whether the stimulation of T7 secretion was specific to the Fe-loaded form of PPIX, we investigated whether the empty (iron-free) protoporphyrin IX (PPIX) or zinc/copper-loaded PPIX could also stimulate EsxA secretion. Fig. 2 shows that only haemin, the Fe-loaded form of PPIX, enhanced secretion of EsxA.

### Ca^2+^ also stimulates T7 secretion

Next we tested whether calcium ions could also regulate secretion. Ca^2+^ is found at millimolar concentrations in mammalian blood and is also highly abundant in pulmonary surfactant [47]. Fig. 1(a) (panel ii) indicates that CaCl_2_ supplementation of TSB medium, at both 2 and 5 mM, increased the level of EsxA in the supernatant. Inclusion of 2 mM CaCl_2_ alongside 1 µM haemin appeared to have additive effects over either supplement alone (Fig. 1a, panels ii and iii). SDS, which has been shown to enhance *essC* mRNA levels [31], did not stimulate EsxA secretion (Fig. 1a, panel iv). Finally, we tested whether either Congo Red or cholesterol, both of which stimulate protein translocation by the type III secretion system [43, 48], could increase EsxA secretion. However, Fig. 1(a) (panel v) indicates that they did not enhance secretion of EsxA and, moreover, Congo Red appeared to downregulate EsxA production.

### T7 secretion is not induced by oxidative stress

In addition to acting as an iron source, at high concentrations haemin induces oxidative damage [49, 50]. To determine whether the haemin-induced hypersecretion of EsxA might be an oxidative stress response, we determined the effect of other oxidative stress agents on EsxA secretion. Fig. S1(a) (available with the online version of this article) shows that in the presence of exogenous hydrogen peroxide there was potentially a small increase in EsxA level in the supernatant. However, in the presence of either diamide or methylviologen (paraquat) there was no stimulation of EsxA secretion and indeed the cellular level of EsxA appeared to be lower than the untreated sample (Fig. S1b, c). Co-supplementation of cultures with 1 µM haemin alongside diamide or methylviologen again resulted in haemin-dependent stimulation of EsxA secretion. We conclude that it is unlikely that the haemin induction of EsxA secretion in strain RN6390 is due solely to oxidative stress.

### Haemin-induced hyper-secretion of EsxA is strain-dependent

**Fig. 3.**
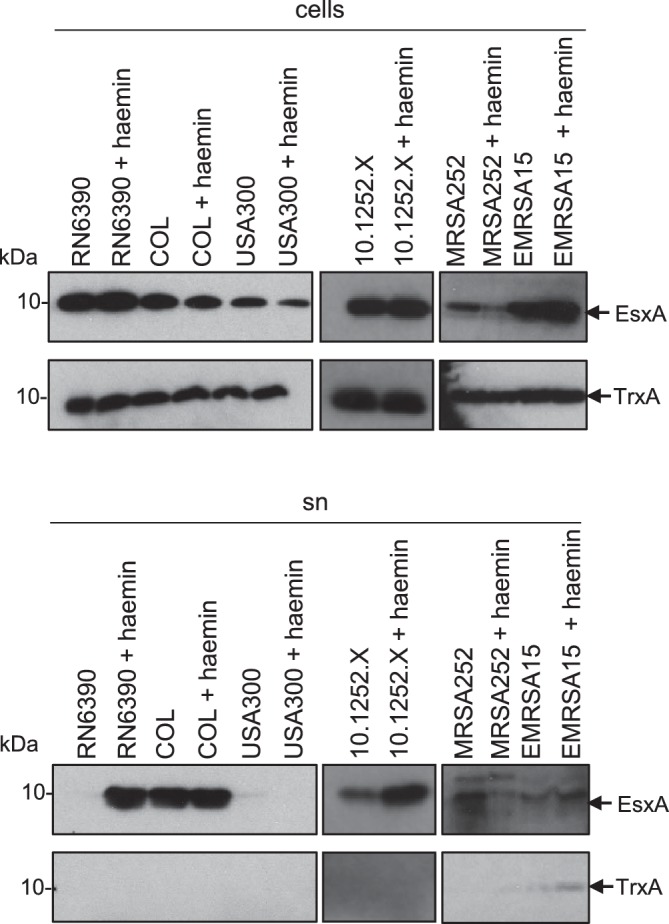
Haemin-induced stimulation of EsxA secretion in *S. aureus* is strain-specific. The indicated *S. aureus* strains were subcultured into TSB medium, or TSB medium supplemented with 4 µM haemin, as indicated, and grown aerobically until an OD_600_ of 2 was reached. Samples were fractionated to give cells and supernatant (sn), and supernatant proteins were precipitated using TCA. For each gel, 4 µl of a cell culture adjusted to an OD_600_ of 1 and 12 µl of culture supernatant were loaded. Western blots were probed with anti-EsxA or anti-TrxA (cytoplasmic control) antisera.

Recent genomic analysis has revealed that there is genetic diversity at the *ess* locus across *S. aureus* strains. The *ess* loci were shown to fall into one of four different groupings, each of which is associated with a specific sequence variant of EssC, and with specific suites of candidate substrate proteins [37]. We therefore undertook experiments to determine whether the haemin-induced stimulation of EsxA secretion was conserved across these groupings. Strain COL is in the same EssC grouping as RN6390 (*essC1*) – both strains belong to the CC8 clonal complex, but are different sequence types (ST8 and ST250, respectively). COL has been noted previously to have a higher level of *in vitro* T7SS activity than RN6390 [25, 29]. Fig. 3 shows that EsxA secretion by COL is indeed higher than that of RN6390, and is comparable to the levels seen when RN6390 is grown with 1 µM haemin. Interestingly, haemin addition to cultures of COL grown in TSB had a negligible effect on the level of EsxA secretion.

We next examined the effect of haemin supplementation on an *essC2*-variant strain, *S. aureus* 10.1252.X. It can be seen (Fig. 3) that 1 µM haemin also had a positive effect on EsxA secretion in this strain. By contrast, when strain MRSA252 (an *essC3* variant) was cultured with haemin, secretion of EsxA was reduced (and there also seemed to be less EsxA associated with the cellular fraction), suggesting a potential repression of *ess* expression in this strain (Fig. 3). Finally when we examined the *essC4* strain variant, EMRSA15, there appeared to be a slight increase of EsxA levels in the supernatant in the presence of haemin, although we noted that there was some cell lysis in this strain as low levels of the cytoplasmic marker protein, TrxA, were also detected in the supernatant fraction. We conclude that the effect of haemin on EsxA secretion is strain-specific but that it clearly enhances secretion in two of the strains we examined.

### Haemin has transcriptional and post-translational effects on the T7SS in RN6390

**Fig. 4.**
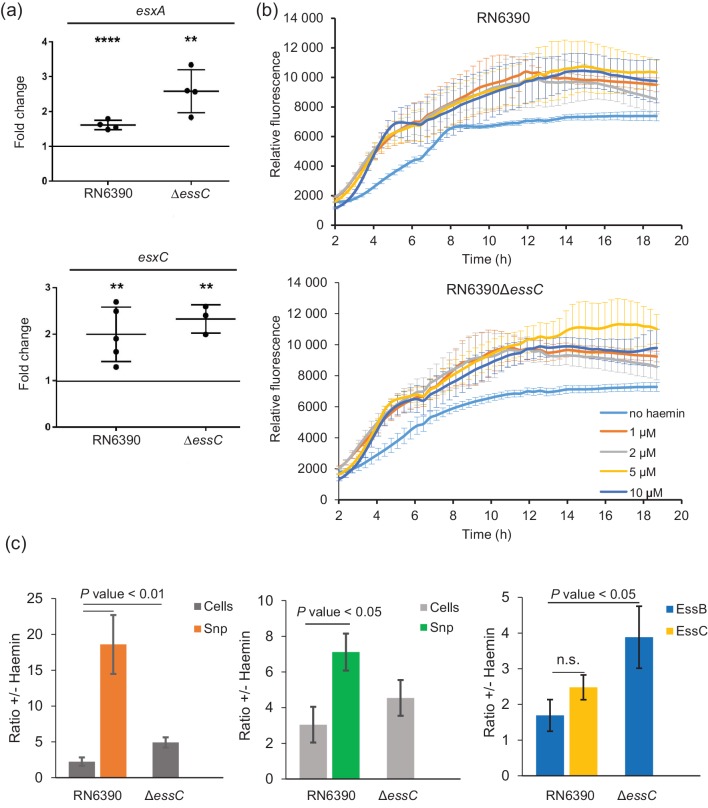
Haemin affects transcription of *esxA* and *esxC* and secretion of the encoded proteins. (a) *S. aureus* RN6390 and the isogenic *essC* deletion strain were grown aerobically in the presence or absence of 1 µM haemin to an OD_600_ of 1, at which point mRNA from at least three biological replicates was prepared as described in the Methods. Relative transcription levels of the *esxA* and *esxC* genes were assayed by RT-qPCR (normalized against the level of 16S rRNA). ****P<0.0001; **P<0.01. (b) *S. aureus* RN6390 and the isogenic *essC* deletion strain were cultured in the presence of the indicated concentrations of haemin for 18 h in 96-well plates (200 µl volume) with shaking. Yellow fluorescent protein (YFP) fluorescence of the culture was monitored at 485 nm and was measured in arbitrary units that were normalized to the growth at each time point. Fluorescence was statistically significantly higher in the presence of haemin than in its absence, for both RN6390 and the *essC* deletion strain (assessed by comparing the relative fluorescence in the absence of haemin with the relative fluorescence measured in the presence of 1, 2, 5 or 10 µM haemin at each time point sampled between 3 and 6 h) (*P*<0.05 in all cases). (c) Quantification of the levels of T7SS-related proteins in the presence and absence of 1 µM haemin. Cultures of RN6390 and the *essC* deletion strain grown aerobically in TSB medium in the presence or absence of 1 µM haemin to an OD_600_ of 2 and separated into cells and supernatant (sn) as described in the Methods. For quantification of protein in the cellular fraction, 5 µl of a cell culture adjusted to an OD_600_ of 1 was loaded and for the TCA-precipitated supernatant an equivalent of 15 µl of culture supernatant was loaded. Quantification results are expressed as the ratio of the signal obtained in the presence 1 µM haemin to that in the absence of haemin. The results represent the mean±sd of at least three biological replicates. *P* values from Student's paired *t*-tests are shown.

We next addressed whether haemin supplementation increased the level of EsxA in the supernatant due to transcriptional upregulation of the *ess* gene cluster. To this end, we isolated mRNA from RN6390 and the isogenic *essC* strain that had been cultured in TSB medium in the presence or absence of 1 µM haemin and used this to prepare cDNA. Since *esxA* is transcribed separately from the other 11 genes at the *ess* locus in RN6390, we undertook RT-qPCR with oligonucleotides designed to separately amplify *esxA* and *esxC*, normalizing against 16S rRNA as an endogenous control. Fig. 4(a) shows that there is a very small, but statistically significant, effect of haemin on both *esxA* and *esxC* transcription in the wild type RN6390 strain (1.5–2-fold). A similar small effect (2–3-fold) was also seen on transcription of these genes in the presence of haemin when the T7SS was inactivated by deletion of *essC*.

To determine whether haemin may also affect translation of the *ess* genes, we constructed a plasmid-encoded fusion of the *esxA* promoter and ribosome binding site with *yfp* and monitored the fluorescence in the same two strains in the presence and absence of exogenous haemin. Fig. 4(b) shows that there is a small (<2-fold) but statistically significant effect of haemin supplementation on YFP fluorescence, consistent with the similar small effect seen on the *esxA* and *esxC* transcript levels by RT-qPCR.

To assess whether haemin had a post-translational effect on T7SS activity in RN6390, we quantified the levels of EsxA and EsxC in the cellular and supernatant fractions for strains grown in the presence and absence of haemin. Fig. 4(c) shows that in the presence of haemin there was a 2–3-fold increase in the cellular level of these two proteins, and a comparable increase in the levels of the membrane-bound T7 components EssB and EssC (representative western blots are shown in Fig. S2). In the *essC* mutant strain, there was also an increase in the cellular level of EssB of approximately 4-fold, and similar increases in the cellular levels of EsxA and EsxC (Fig. 4b). These small but significant increases in cellular levels of protein mirror the similar small increases in transcription of these genes in the presence of haemin. However, analysis of the supernatant fractions shows that in the presence of haemin, there was a 15–20-fold increase in the amount of EsxA in the culture supernatant and a 6–8-fold increase in the supernatant levels of EsxC. These findings are consistent with haemin having a post-translational effect on the secretion activity of the T7SS in strain RN6390. Alternatively, it is possible that haemin affects the levels of extracellular proteases such that the accumulation of EsxA and EsxC in the culture supernatant when haemin is present is an indirect effect due to decreased turnover of these proteins.

### Inactivation of RN6390 *essC* mounts an iron starvation transcriptional response

Taken together, the results so far indicate that haem iron has a striking effect on the secretion activity of the T7SS in *S. aureus* strain RN6390, potentially implicating the secretion system in iron homeostasis. To investigate this further, we examined differences in the transcriptional profile between wild type reference strain RN6390 and an isogenic *essC* deletion mutant. Total RNA was prepared from exponentially growing cultures as described in the Methods and RNA-seq was used to investigate gene expression levels. As shown in Table 2 and Fig. S3, a group of 41 genes displayed at least a log 2-fold statistically significant change in expression in an *essC* mutant, with seven being down-regulated (excluding *essC* itself) and 34 being up-regulated. Interestingly, 25 of the up-regulated genes have known or implied roles in iron acquisition by *S. aureus* [51], and these are listed in Table S1. Also included in Table S1 are the fold changes for all of the other genes involved in these iron acquisition pathways but which did not reach our log 2-fold cut-off.

**Table 2.** Genes differentially regulated (>log 2-fold) in the RN6390 *essC* deletion mutant, sorted by ascending fold change

**Locus ID**	**Gene name**	**Fold change**	**Description**	**Known regulators**
Downregulated genes				
SAOUHSC_00262	*essC*	−29.4	T7SS ATPase EssC	
SAOUHSC_02290	–	−7.8	Unknown, hypothetical protein	
SAOUHSC_01942	*splA*	−5.4	Highly specific serine protease specific to *S. aureus*	Agr (indirect) [60]
SAOUHSC_01944	–	−4.5	Unknown, hypothetical protein	
SAOUHSC_02243	*lukG*	−4.5	Leukocidin-like toxin	
SAOUHSC_01941	*splB*	−4.3	Serine protease SplB	Agr [60]
SAOUHSC_01938	*splD*	−4.3	Serine protease SplD	Agr [60]
SAOUHSC_01121	*hla*	−4.1	α-Haemolysin	Agr [61]
Upregulated genes				
SAOUHSC_02433	*sfaC*	4.1	Unknown, hypothetical protein	Fur [62]
SAOUHSC_02865	*feoA*	4.4	FeoA domain-containing protein	
SAOUHSC_00653	*fhuB*	4.5	Ferrichrome transport permease FhuB	Fur [63]
SAOUHSC_02653	–	4.6	Putative Gcn5-related *N*-acetyltransferase domain profile	
SAOUHSC_02434	*sfaB*	4.6	Putative siderophore biosynthesis protein	Fur [62]
SAOUHSC_00071	*sirC*	4.6	Involved in staphyloferrin B transport into the cytoplasm	Fur [64]
SAOUHSC_00246	–	4.7	Putative transmembrane efflux pump protein	
SAOUHSC_01089	*isdG*	4.7	Haem-degrading monooxygenase IsdG	Fur [65]
SAOUHSC_00245	–	5.2	Putative transposase	
SAOUHSC_02428	*htsB*	5.4	Haem transport system permease HtsB	Fur [66]
SAOUHSC_01081	*isdA*	5.4	Iron-regulated haem-iron binding protein	Fur [65]
SAOUHSC_02719	–	5.5	ABC transporter ATP-binding protein	
SAOUHSC_01082	*isdC*	5.5	Haem transporter IsdC	Fur [65]
SAOUHSC_02654	*trxB2*	5.5	Thioredoxin reductase TrxB2	
SAOUHSC_01085	*isdE*	5.6	Haem-receptor lipoprotein IsdE	Fur [65]
SAOUHSC_00130	*isdI*	5.7	Haem-degrading monooxygenase IsdI	
SAOUHSC_01086	*isdF*	6.1	ABC permease IsdF	Fur [65]
SAOUHSC_00131	–	6.1	Putative membrane spanning protein	
SAOUHSC_01088	*srtB*	6.2	Sortase SrtB	Fur [65]
SAOUHSC_01084	*isdD*	6.2	ATP-hydrolysing and haem-binding protein IsdD	Fur [65]
SAOUHSC_02432	–	6.2	Unknown, hypothetical protein	
SAOUHSC_01087	–	6.3	Iron compound ABC transporter permease	
SAOUHSC_02655	–	6.3	Unknown, hypothetical protein	
SAOUHSC_02245	–	6.5	Unknown, hypothetical protein	
SAOUHSC_02435	*sfaA*	6.7	Putative transporter	
SAOUHSC_02554	*fhuD2*	6.8	Ferric hydroxamate receptor 1 FhuD2	Fur [67]
SAOUHSC_00652	*fhuA*	7.0	Ferrichrome ABC transporter ATP-binding protein FhuA	Fur [63]
SAOUHSC_00072	*sirB*	7.4	Involved in staphyloferrin B transport into the cytoplasm	Fur [64]
SAOUHSC_02246	*fhuD1*	8.0	Iron compound ABC transporter FhuD1	Fur [68]
SAOUHSC_00747	*sstB*	9.0	Ferrichrome ABC transporter permease SstB	Fur [69]
SAOUHSC_00748	*sstC*	9.1	Ferrichrome ABC transporter ATP-binding protein SstC	Fur [69]
SAOUHSC_02430	*htsA*	10.5	Haem transport system lipoprotein HtsA	Fur [66]
SAOUHSC_00746	*sstA*	10.9	Ferrichrome ABC transporter permease SstA	Fur [69]
SAOUHSC_00074	*sirA*	16.3	Receptor component of staphyloferrin B	Fur [64]

It is apparent that many of the genes that encode the Isd machinery, which is involved in haem acquisition, are upregulated. Furthermore, genes for the biosynthesis and uptake of the two *S. aureus* siderophores, staphyloferrin A and staphyloferrin B, are also upregulated, as are genes encoding the Fhu machinery, which *S. aureus* uses to import xenosiderophores produced by other bacteria [51]. All of these genes are known to be regulated by the ferric uptake regulatory (Fur) protein (Table 2). These findings indicate that inactivation of the T7SS by deletion of the *essC* gene prompts *S. aureus* RN6390 to mount an iron starvation response.

### The *S. aureus* RN6390 and *essC* strains grow similarly under iron limitation

**Fig. 5.**
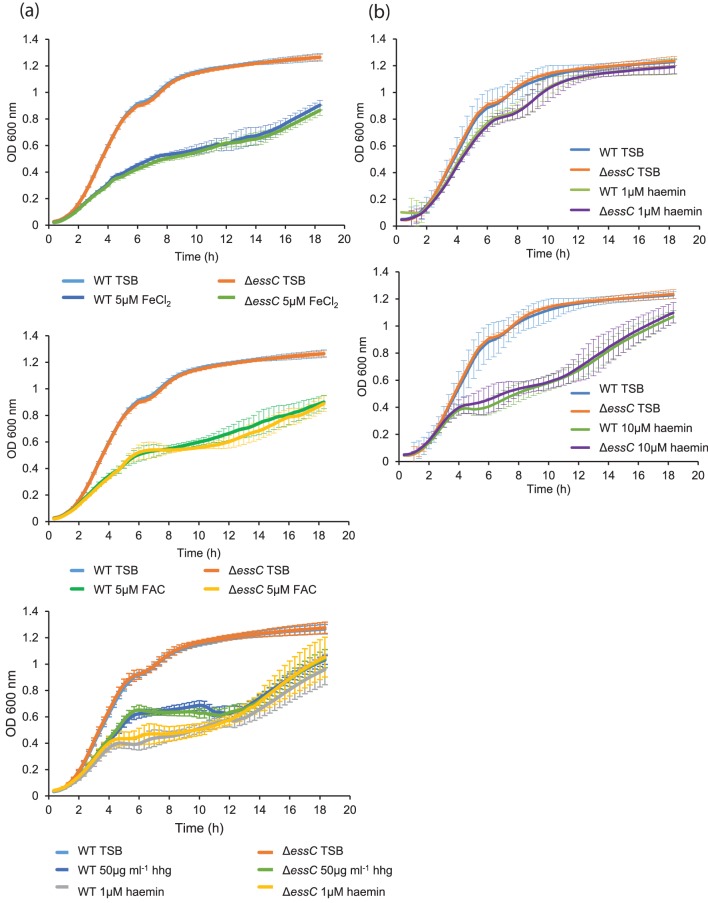
Effect of iron limitation and haemin on growth of the *S. aureus* RN6390 and *essC* mutant strains. (a) *S. aureus* RN6390 and the *essC* deletion strain were grown with shaking in either TSB medium or iron-depleted TSB medium (prepared using Chelex-100 to remove all divalent cations followed by re-introduction of Zn^2+^, Mn^2+^, Mg^2+^ and Ca^2+^ as described in the Methods) that had been supplemented with the indicated iron source. FAC, ferric ammonium citrate; hhg, human haemoglobin. (b) Growth of *S. aureus* RN6390 and the *essC* deletion strain in TSB or TSB supplemented with 1 or 10 µM haemin, as indicated. Growth was monitored over 18 h in 96-well plates (200 µl volume). Error bars are ±sd, *n*=3.

Since RNA-seq analysis suggested that the *essC* strain was iron-starved, we next investigated the growth behaviour of strain RN6390 and the *essC* derivative under iron-limited growth conditions. To this end, we used a strong divalent cation chelator to remove iron and other divalent cations from TSB growth medium, and added back the essential cations Zn^2+^, Mn^2+^, Mg^2+^ and Ca^2+^ [33, 52]. Growth of both the wild type and the Δ*essC* strains reached a plateau at low optical density, indicating that growth was impaired, presumably because iron had now become a limiting factor (Fig. S4). Next we supplemented the growth medium with 50 μg human haemoglobin ml^−1^, 5 µM ferrous chloride or ferric ammonium citrate, or 10-fold dilutions of haemin from 10 µM to 1 nM (Figs 5a and S4). Addition of iron sources clearly stimulated growth of both strains, although growth of the *essC* strain was almost indistinguishable from that of the parental strain regardless of the source of iron (Fig. 5a) or the concentration of haemin added (Fig. S4). We conclude that there is no evidence that the *essC* strain is impaired in iron acquisition.

Finally, since haem is toxic to *S. aureus* at high concentrations (5–10 µM; [49]), we assessed whether the *essC* mutant was more sensitive to haem toxicity. Haemin was clearly toxic for strain RN6390, since even at 1–2 µM growth was slower than in the absence of haemin (Figs 5b and S5). However, the *essC* mutant showed similar growth kinetics to the parental strain for all of the haemin concentrations tested, and is therefore not measurably more sensitive to haemin toxicity.

### Concluding remarks

Here we have identified a number of links between the T7SS of *S. aureus* strain RN6390 and iron. We have shown that haemin affects T7 secretion at a transcriptional level and also significantly enhances the level of secreted substrates. Furthermore, inactivation of the essential T7 secretion gene, *essC*, resulted in upregulation of a large group of Fur-regulated genes that are required for iron acquisition. In this context, it is interesting to note that the ESX-3 T7SS from both *Mycobacterium tuberculosis* and *Mycobacterium smegmatis* plays a key role in iron homeostasis, under iron-replete and iron-sufficient conditions, and secretes at least two substrates involved in siderophore-mediated iron uptake [10, 53]. However, despite these observations the *essC* mutant strain was not phenotypically iron-starved, nor was it more sensitive to haemin toxicity. Unravelling the links between the *S. aureus* T7SS and iron homeostasis will require further analysis.

## Supplementary Data

754 Supplementary File 1
