# Supplementary material for: Haem-iron plays a key role in the regulation of the Ess/type VII secretion system of Staphylococcus aureus RN6390
Source: Microbiology (Reading). 2017 Nov 24;163(12):1839–50. doi: 10.1099/mic.0.000579 (PMC5845736; doi:10.1099/mic.0.000579)
Supplement: Supplementary File 1 [file mic-163-1839-s001.pdf]

Heme-iron plays a key role in the regulation of the Ess/Type VII secretion system of *Staphylococcus aureus* RN6390

SUPPLEMENTARY INFORMATION

M. Guillermina Casabona[1¥], Holger Kneuper[1¥], Daniela Alferes de Lima[1] Catriona P. Harkins[2], Martin Zoltner[1], Erik Hjerde[3], Matthew T.G. Holden[2] and Tracy Palmer[1*]

[1]Division of Molecular Microbiology School of Life Sciences, University of Dundee, Dundee, UK.

[2]School of Medicine, University of St Andrews, St Andrews, KY16 9TF, UK.

[3]Department of Chemistry, Arctic University of Norway, Tromsø, Norway

[¥] Contributed equally to this work. Listed alphabetically.

*To whom correspondence should be addressed.

Tel +44 1382 386464

e-mail t.palmer@dundee.ac.uk

**Heme acquisition:**
isdA (5.4 fold), *isdB* (2.1*), *isdC* (5.5 fold), *isdD* (6.2 fold), *isdE* (5.6 fold), *isdF* (6.1 fold), *isdG* (4.7 fold), *isdH* (1.8*), *isdI* (5.7 fold), *srtB* (6.2 fold), *SAOUHSC_00131* (6.1 fold), *SAOUHSC_01087* (6.3 fold).
**Staphyloferrin A biosynthesis and uptake:**
sfaA (6.7 fold), *sfaB* (4.6 fold), *sfaC* (4.1 fold), *sfaD* (1.8 fold), *htsA* (10.5 fold), *htsB* (5.4 fold), *htsC* (1.7 fold).
**Staphyloferrin B biosynthesis and uptake:**
sbnA (1.9 fold), *sbnB* (1.9 fold), *sbnC* (3.5 fold), *sbnD* (1.7 fold), *sbnE* (1.6 fold), *sbnF* (1.5 fold), *sbnG* (1.4 fold), *sbnH* (2.5), *sbnI* (2.3*), *sirA* (16.3 fold), *sirB* (7.4 fold), *sirC* (4.6 fold).
**Ferrichrome import**
fhuA (7.0 fold), *fhuB* (4.5 fold), *fhuG* (1.6 fold), *fhuD1* (8.0 fold), *fhuD2* (6.8 fold), *sstA* (10.9 fold), *sstB* (9.0 fold), *sstC* (9.1 fold) *sstD* (3.2 fold), *SAOUHSC_02245* (6.5 fold).

Table S1. Genes involved in iron acquisition and level of upregulation in the *essC* mutant relative to wild type. *genes for which p value > 0.05 but were included for completeness.

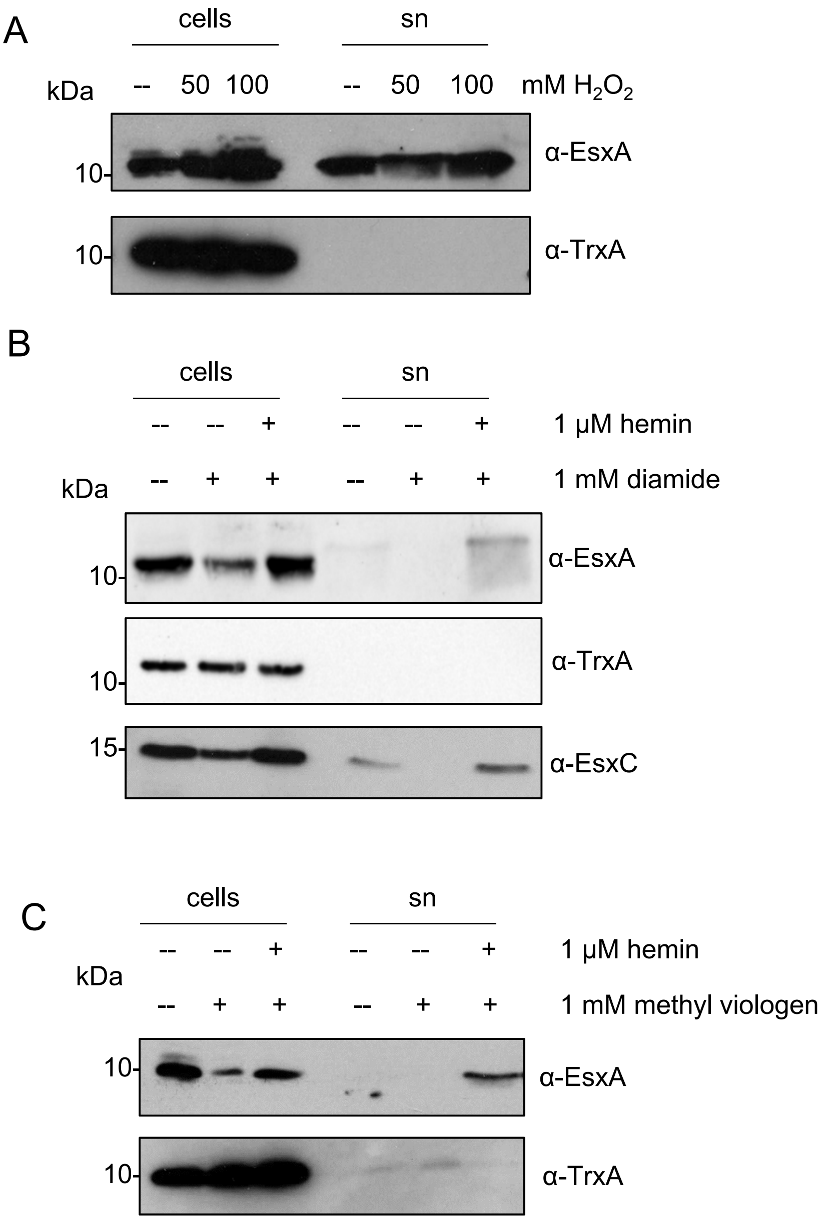

Figure S1. Effect of oxidative stress on EsxA secretion. *S. aureus* RN6390 was grown in the presence of the indicated concentrations of (A) H_2O_2, (B) diamide, or (C) methylviologen with or without the additional inclusion of hemin, and the secretion of EsxA was assessed by western blotting. For each gel, 5 µl of OD_{600} 1 adjusted cells and an equivalent of 15 µl of culture supernatant (sn) were loaded. Western blots were probed with anti-EsxA, anti-EsxC or anti-TrxA (cytoplasmic control) antisera.

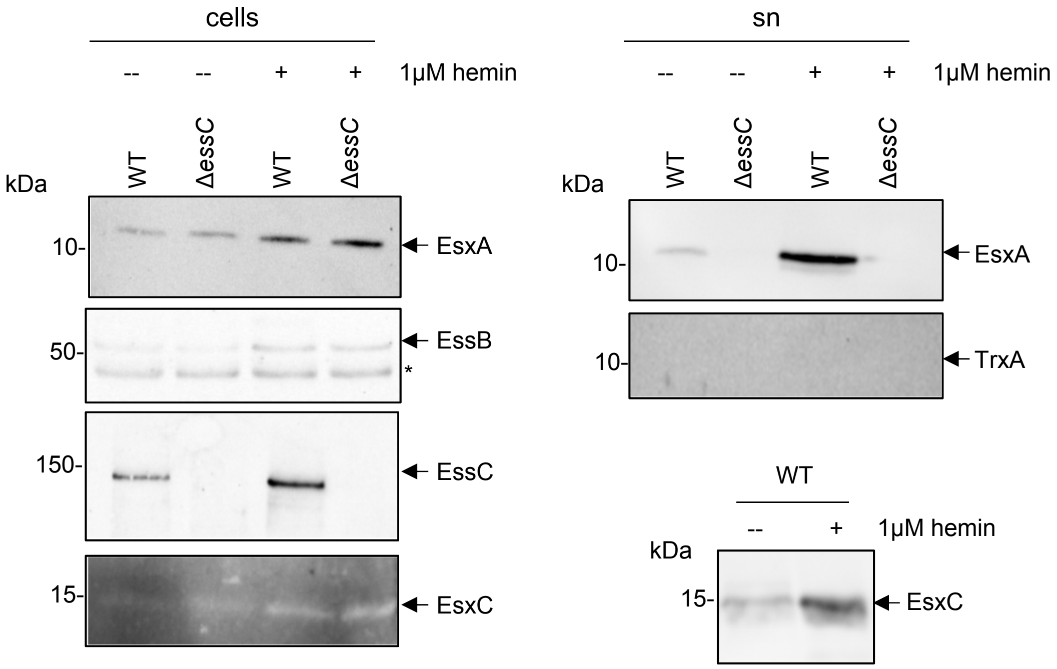

Figure S2. Levels of T7 components and substrates in the presence or absence of hemin. RN6390 and the isogenic Δ*essC* mutant were grown aerobically in TSB medium in the presence or absence of 1 μM hemin until OD_{600} of 2 was reached, after which samples were separated to give cells and supernatant. Representative western blots of EsxA and EsxC in cells and in TCA-precipitated supernatants (sn), and of EssB and EssC in cells are shown. The TCA-precipitated supernatants were also probed with anti-TrxA antisera to detect any cytoplasmic leakage. For quantification of protein in the cellular fraction 5 μl of OD_{600} 1 adjusted cells was loaded and for the TCA-precipitated supernatant an equivalent of 15 μl of culture supernatant was loaded. * indicates an unspecific band detected by the anti-EssB antiserum.

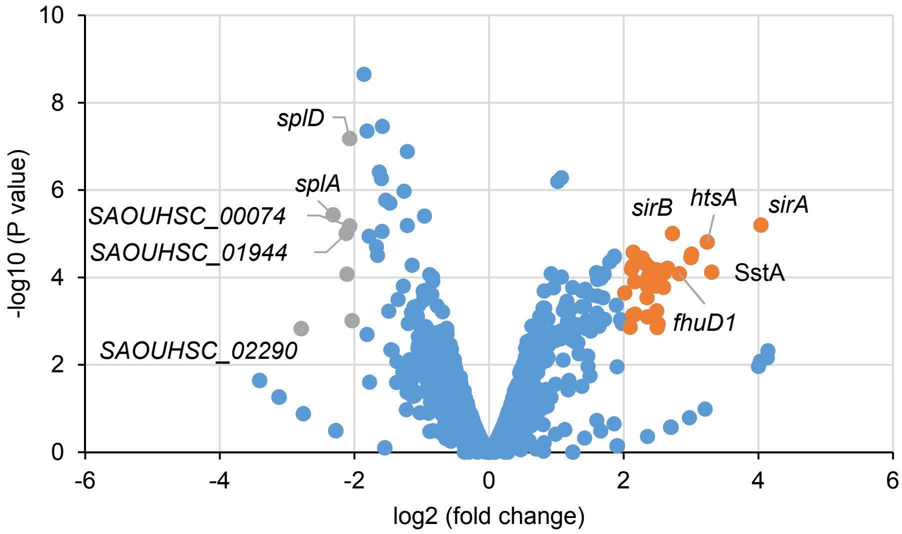

Figure S3. Volcano plot representation of the differentially expressed genes in RN6390 strain compared to the isogenic *essC* mutant. The orange and grey spots represent, respectively, genes that are up- or down-regulated in *essC* mutant relative to the parental strain. Note that the *essC* gene was removed from this analysis.

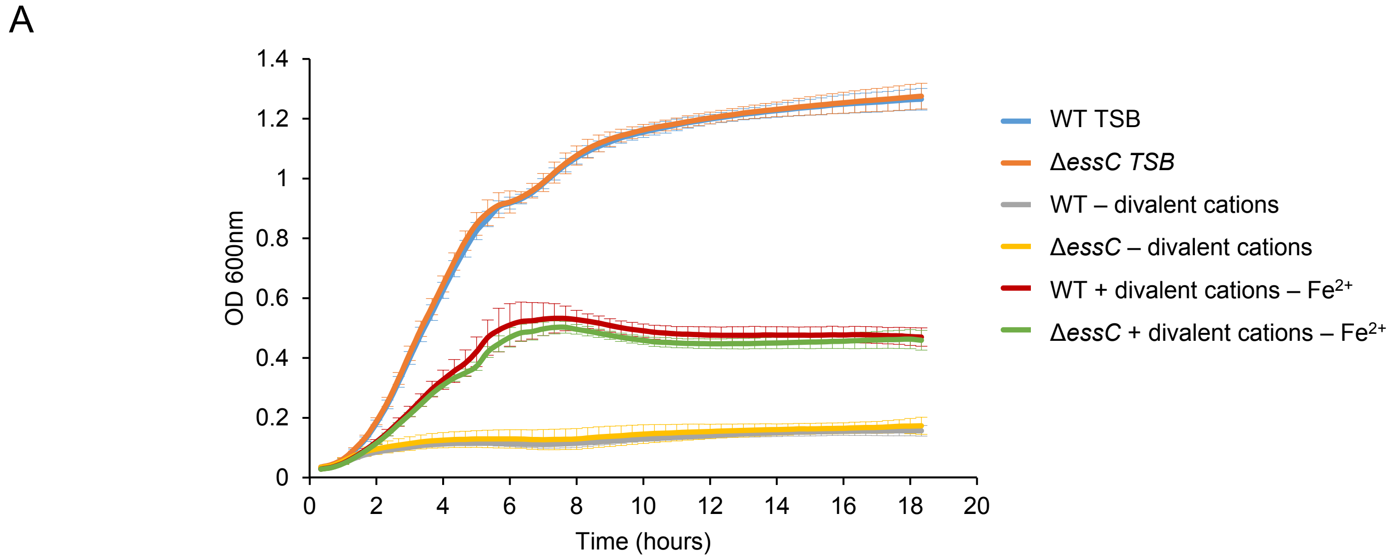

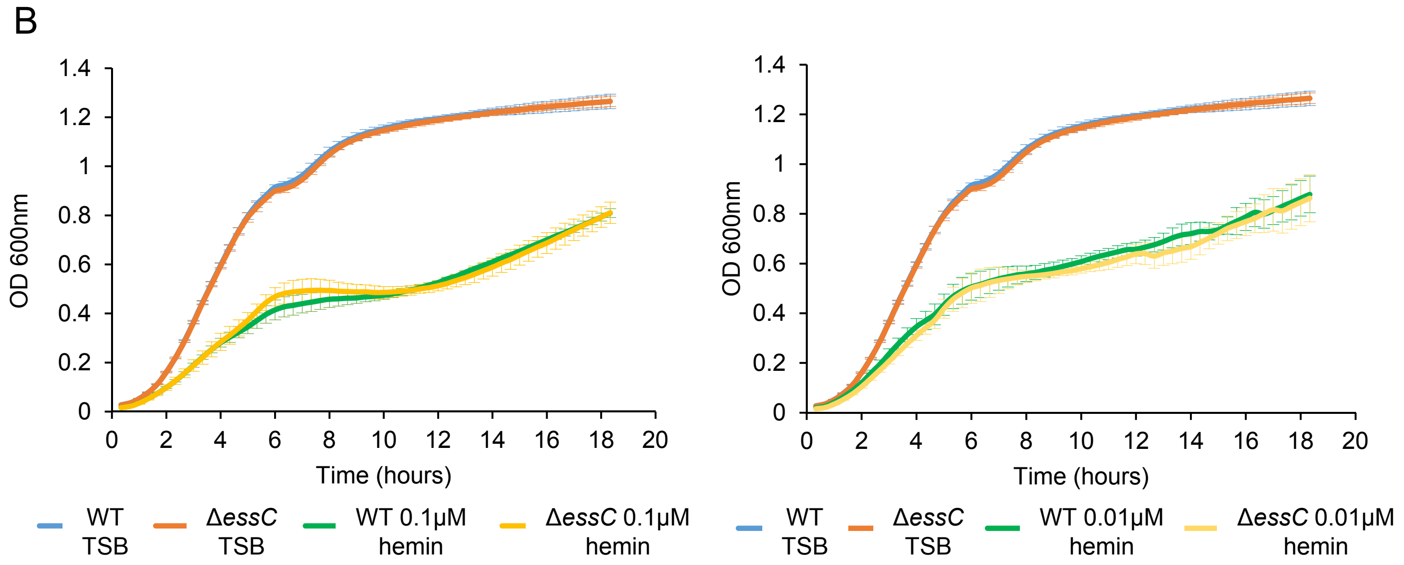

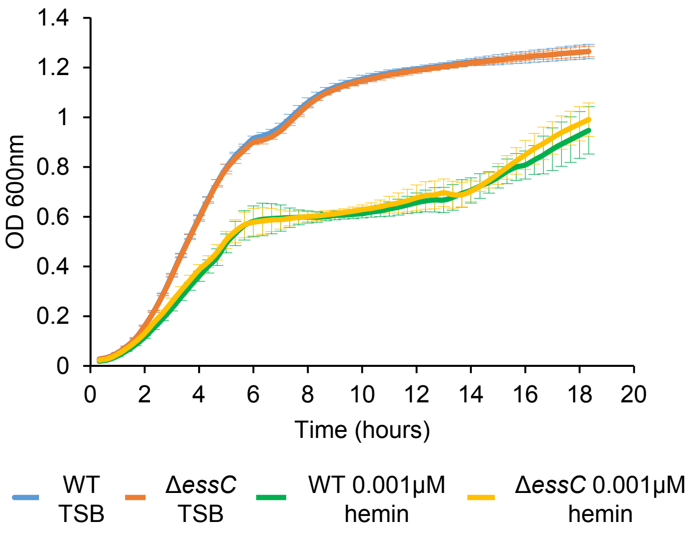

Figure S4. Effect of iron and divalent cation depletion on the growth of *S. aureus* RN6390. (A) *S. aureus* RN6390 and the isogenic *essC* deletion strain were grown with shaking in either TSB medium, TSB medium depleted for divalent cations (by treatment with Chelex-100) or depleted TSB medium that had been supplemented with a cocktail of 25 µM $ZnCl_2$, 25 µM $MnCl_2$, 1mM $MgCl_2$ and 100 µM $CaCl_2$ (annotated as + divalent cations – Fe^{2+}). (B) *S. aureus* RN6390 and the isogenic *essC* deletion strain were grown in Chelex-100 treated TSB medium supplemented with divalent cations and the indicated different concentrations of hemin as iron source. Growth was monitored over 18 hours in 96-well plates (200 µl volume). Error bars are ± standard deviation, *n*=3.

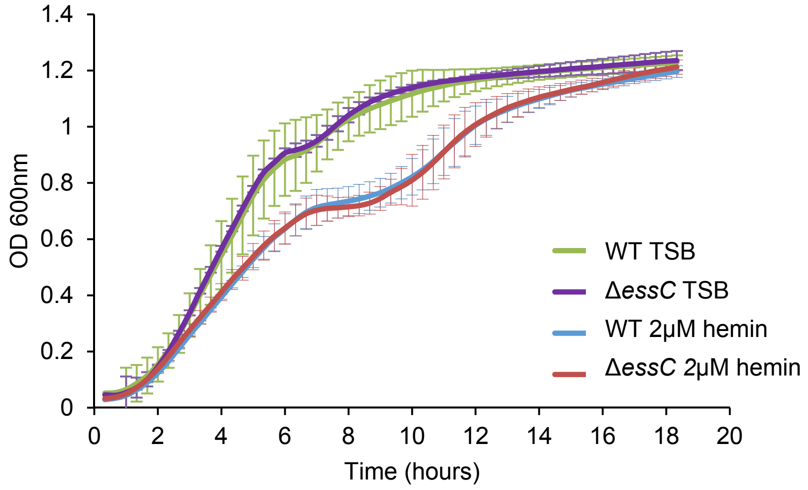

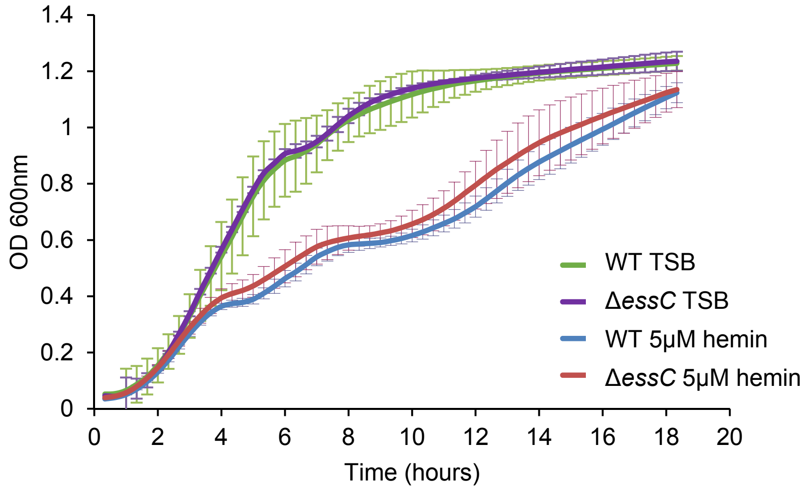

Figure S5. Growth of *S. aureus* RN6390 and *essC* mutant strains with micromolar levels of hemin. Strains RN6390 and the isogenic *essC* deletion were grown with shaking in TSB medium or TSB medium supplemented with 2 μM or 5 μM hemin, as indicated. Growth was monitored over 18 hours in 96-well plates (200 μl volume). Error bars are ± standard deviation, *n*=3.
